# Long-Term Outcomes Following Surgical Intervention for Achilles Tendon Rupture: A Systematic Review With a Minimum Five-Year Follow-Up

**DOI:** 10.7759/cureus.77614

**Published:** 2025-01-18

**Authors:** Conor J Kilkenny, Gordon Daly, Shane Irwin, Tom Doyle, Anoushka R Saldanha, Lama M Alghawas, Niall McGoldrick, John Quinlan

**Affiliations:** 1 Orthopaedics, Tallaght University Hospital, Dublin, IRL; 2 Surgery, Royal College of Surgeons in Ireland, Dublin, IRL; 3 Orthopaedics, Royal College of Surgeons in Ireland, Dublin, IRL; 4 Orthopaedics, Galway University Hospital, Galway, IRL; 5 Orthopaedics, Trinity College Dublin, Dublin, IRL; 6 Trauma and Orthopaedics, Tallaght University Hospital, Dublin, IRL

**Keywords:** achilles rupture, complications, operative management, tendon, trauma

## Abstract

Achilles tendon (TA) rupture is a common injury. However, there is limited evidence on the long-term outcomes following surgical repair. This systematic review aims to evaluate the efficacy and complications associated with surgical management of TA rupture with a minimum five-year follow-up duration.

A literature search of Medline OVID, EMBASE, and Cochrane databases was performed adhering to Preferred Reporting Items for Systematic Reviews and Meta-Analyses (PRISMA) guidelines. The outcomes measured focused on postoperative complications, rates of re-rupture, contralateral rupture, and revision surgery.

Six studies including 806 patients were included. Surgical repair demonstrated low re-rupture rates (4.2%) and revision rates (3.7%) at five years follow-up. Contralateral ruptures were uncommon (N=5), and 30 patients required revision surgery. However, complications such as deep vein thrombosis (6.3%) and wound infections (3.6%) were observed.

Surgical repair of TA rupture demonstrates excellent long-term outcomes, including low rates of re-rupture and revision over a minimum of five years. While complications such as deep vein thrombosis and wound infections are observed, this systematic review highlights the reliability, durability, and overall success of surgical intervention in effectively treating this condition.

## Introduction and background

Achilles tendon (TA) rupture remains a clinical challenge, which has a significant impact on patient morbidity and functional outcomes. The incidence of TA rupture has increased in recent years [1], underlining the need for effective interventions and long-term outcome assessments. TA rupture appears to be more prevalent among athletes and the increasingly active aging population [1-3]. Surgical intervention for acute TA rupture has previously shown a reduced risk of re-rupture when compared with non-operative treatment [4-7]. However, there have been very few studies, which have assessed the long-term outcome of patients following surgical repair.

Surgical repair is commonly utilized in the management of TA rupture, aiming to restore anatomic length and physiologic tension, while providing adequate strength to aid in return to activity [8]. There are also various surgical techniques for treating a TA rupture, which vary from open repair to minimally invasive approaches, with each technique associated with its own potential benefits and drawbacks [9-11]. Surgical intervention has been shown to reduce the risk of re-rupture; however, an increased rate of other complications is seen when compared to conservative management [12,13]. Therefore, the decision-making process regarding surgical intervention is not straightforward, with careful consideration of the benefits and risks associated with surgical intervention required. This systematic review examines the outcomes and complications associated with surgical repair, an understanding of which is essential for both clinicians and patients when making decisions regarding TA rupture repair.

TA repair is a commonly performed surgical procedure. However, there is an evident gap in our understanding of the long-term outcomes for patients. The short-term results following surgical repair are well documented in the literature. The paucity of studies in the literature that focus on the long-term outcomes following surgical repair leads to potential questions regarding the durability and effectiveness of surgical intervention with respect to long-term outcomes. The purpose of this study is to evaluate the long-term outcomes of surgical repair following TA rupture in studies with a minimum five-year follow-up.

## Review

Methods

Search Strategy

An electronic search was performed on Medline OVID, EMBASE, and Cochrane databases on 3 November 2023 for relevant studies, in accordance with the Preferred Reporting Items for Systematic Reviews and Meta-Analyses (PRISMA) guidelines. The study protocol was devised by two independent reviewers (CJK and GRD) The search was performed by two independent reviewers (GRD + CJK) using a pre-determined search strategy. Search terms included: (‘Achilles tendon’) AND (‘surgery’) AND (‘outcomes’) AND (‘complications’). The full search summary can be found in Table 4 of Appendices. After the manual removal of duplicates, all titles and abstracts were independently screened by CJK and GRD. Potentially eligible studies then underwent full-text review for inclusion using predetermined criteria. A third reviewer (LA) arbitrated in instances of disagreement. Reference lists of included studies were then screened for additional suitable studies.

Eligibility Criteria

The inclusion criteria were the following: (1) clinical study on TA rupture, (2) minimum five-year follow-up, (3) operative management, (4) rate of postoperative complications reported, (5) rate of re-rupture reported, (6) rate of revision surgery reported, (7) published in a peer-reviewed journal, and (9) published in English.

The exclusion criteria were the following: (1) review studies, (2) case reports, (3) literature reviews and meta-analyses, (4) cadaveric studies, (5) biomechanical studies, and (6) conference abstract only.

Data Extraction and Quality Assessment

Two independent reviewers (CJK and GRD) extracted characteristic data including author and publication name, number of patients included, sex of patients, level of evidence (LOE) of each study, study design, and mean age of patients included, using a pre-defined electronic spreadsheet. The LOE was evaluated based on the guidelines of the *Journal of Bone and Joint Surgery* [14]. The MQOE was evaluated by use of a modified Coleman methodology score [15]. Studies were considered excellent quality if they scored 85-100, good quality if they scored 70-84, fair quality if they scored 55-69, and poor quality if they scored less than 55.

Outcomes recorded were (1) postoperative complications, (2) rates of re-rupture, (3) revision surgery rates, and (4) contralateral rupture rates.

Results

**Figure 1:**
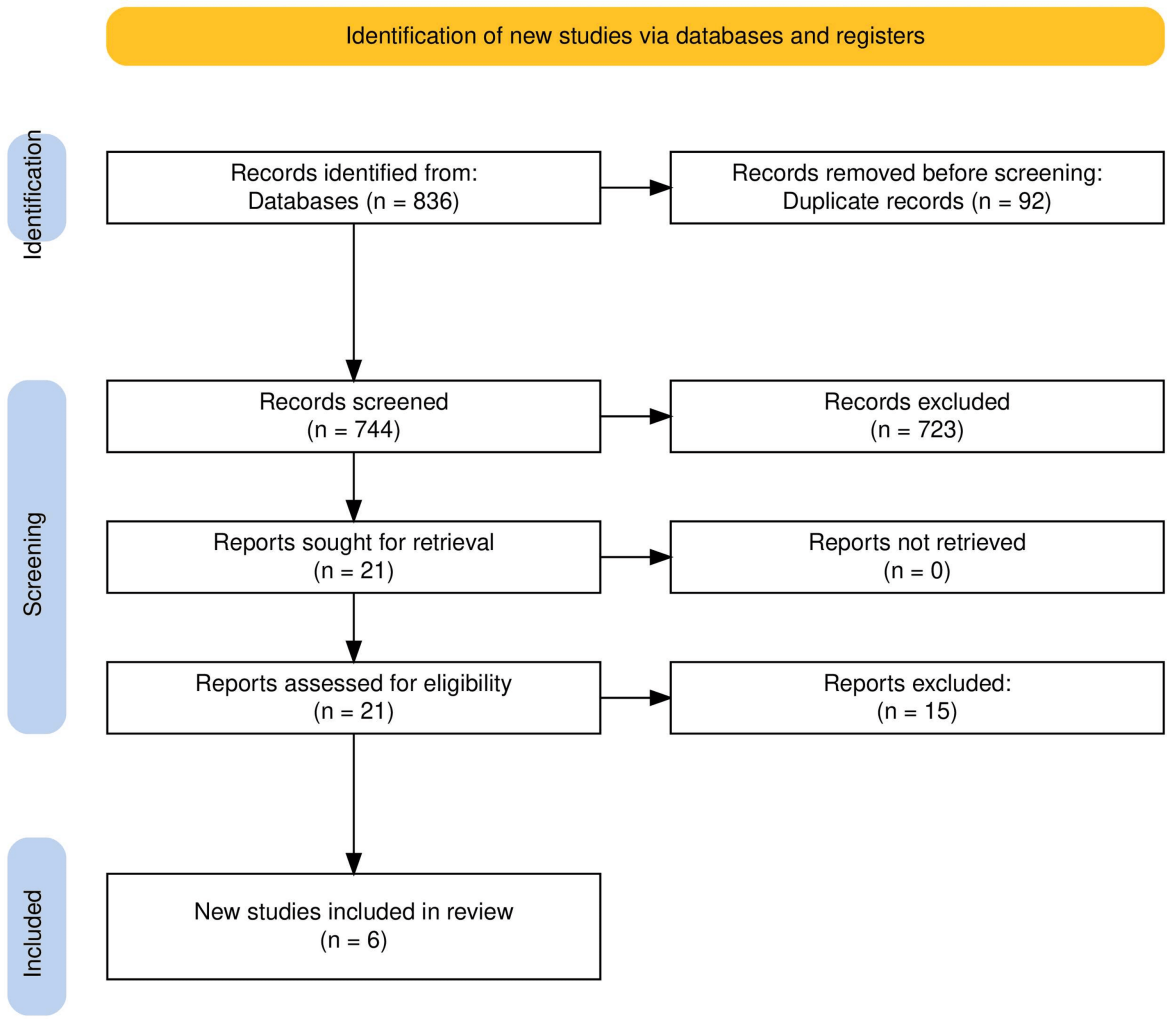
Prisma flow diagram

The study selection flowchart is presented in Figure 1.

A total of 806 patients were included according to our criteria. 676 were male, while 130 were female. The mean age overall was 39.5 years. The mean Coleman score was 68.5 indicating the studies included were considered a "fair quality" of evidence. Detailed demographic data can be found in Table 1.

**Table 1:** Study characteristics and patient demographics LOE, level of evidence; TA, Achilles tendon

Study characteristics and patient demographics										
Lead author	Title	Year	LOE	Coleman	Patient no.	Male	Female	Mean age (years)	Study design	Type of surgery
Fell et al [16]	Surgical repair of acute Achilles tendon ruptures: a follow-up of 639 consecutive cases	2020	II	75	631	529	102	39.2	Long-term prospective cohort study	Open end-to-end repair-Modified Kessler suture
Heikkinen et al. [17]	Augmented compared with nonaugmented surgical repair after total Achilles rupture	2016	I	82	55	48	7	38	Long-term follow-up of previous prospective randomized controlled trials	Simple end-to-end non-augmented Krakow locking loop repair vs augmented fascial flap repair
Kerkhoffs et al. [18]	Functional treatment after surgical repair of acute Achilles tendon rupture: wrap vs walking cast	2002	II	61	39	32	7	36.3	Quasi-randomized trial	Open surgery, three-tissue bundle technique
Lantto et al. [19]	Early functional treatment versus cast immobilization in tension after Achilles rupture repair: results of a prospective randomized trial with 10 or more years of follow-up	2015	I	82	37	34	3	36	Randomized control trial	Modified two-suture Kessler technique; gastrocnemius aponeurosis flap turned down over the suture line and stitched to the TA
Mavrodontidis et al. [20]	Percutaneous repair of acute Achilles tendon rupture: a functional evaluation study with a minimum 10-year follow-up	2015	IV	39	11	11	0	39.3	Retrospective cohort	Percutaneous repair
Maempel et al. [21]	Operative repair of acute Achilles tendon rupture does not give superior patient-reported outcomes to non-operative management	2020	I	72	33	22	11	56	Randomized controlled trial	Open repair core Kessler stitch and interrupted circumferential sutures

The complication rates reported in this study can be seen in Table 2. All studies reported complications. If a study did not report on a specific complication, it was assumed it did not occur during the follow-up period. Deep vein thrombosis (DVT) was the most common complication found postoperatively at 6.3%.

**Table 2:** Complications

Outcome	No. of studies	Result (%, N/806)
DVT	1	6.3%, 51/806
Infection	4	3.6%, 29/806
Delayed wound healing	1	0.2%, 2/806
Sensory impairment/nerve injury	3	1.1%, 9/806
Stiffness	1	0.1%, 1/806
Acquired adjusted shoes post-op	1	0.4%, 3/806
Muscle atrophy	1	0.9%, 7/806

A total of 34 patients suffered a re-rupture during the minimum five-year follow-up period, with five patients suffering a contralateral TA rupture. Thirty patients underwent revision during the follow-up period (Table 3). One study stated the overall rate of contralateral rupture but did not specify whether this occurred in operatively or non-operatively managed patients and thus was not included in the analysis [21].

**Table 3:** Rates of re-rupture/revision

Outcome	No. of studies	Result (%, N/806)
Re-rupture	5	4.2%, 34/806
Contralateral rupture	2	0.6%, 5/773
Revision	3	3.7%, 30/806

Discussion

The management of TA rupture through surgical interventions is a continually evolving field. The most important finding of this review is that excellent clinical outcomes were reported following surgical repair for TA rupture at long-term follow-up, including low re-rupture rates (4.2%) and low rates of revision (3.7%). However, this study found moderate rates of other complications, particularly DVT and wound infections postoperatively.

There is currently no agreed standard of care for patients following TA rupture. There appears to be a move toward minimally invasive techniques in an attempt to provide an alternative to the traditional open repair technique and its potential-associated complications [22,23]. In addition, non-operative management strategies such as platelet-rich plasma (PRP) and stem cells are gaining interest due to their potential to stimulate tendon healing as well as avoid surgical complications [24-26]. The timing of surgery post-injury is another subject of ongoing research, with some advocating for delayed intervention to allow soft tissue swelling to subside [27-29].

A very significant finding of this study is the low re-rupture rate at long-term follow-up. This is an encouraging finding for patients and demonstrates the durability associated with surgical repair. Many other systematic reviews on this area have demonstrated the superior outcomes of surgical repair with respect to re-rupture rates [7,30,31]. Non-operative rates of re-rupture have been found to vary between 3.9% and 13% [31]. However, this is the first systematic review, to our knowledge, to study this over a prolonged period. Re-rupture rates in this study are consistent with previous studies, which found rates of 3.5% to 4.3% [5,30,32]. However, a recent systematic review performed by Shoap et al. has shown even lower rates of re-rupture following surgical management [33]. Shoap et al. found that re-rupture rates were 2.11% and 2.62% following open and minimally invasive surgery, respectively [33]. The mean follow-up in Shoap et al.’s study was 23.45-28.14 months for open and minimally invasive surgery, respectively [33]. The fact this study has similar rates of re-rupture is encouraging given the longer period of follow-up.

Complication rates following surgical repair of TA ruptures are reported within the literature between 4.9% and 34.1% [31]. Thus, the complication rates in our study are consistent with previous literature. High rates of postoperative DVT and wound infections reported in this study are also consistent with many studies [6,12]. Sural nerve injury is a known risk associated with surgical repair of the TA [34,35]. However, the rates of nerve injury in this study appear to be lower than in other systematic reviews [30,32].

The results of this study are very promising for patients who receive surgical management; however, various surgical techniques are described in this study. Different surgical techniques have their own innate advantages and potential drawbacks. Many studies have compared open vs. minimally invasive surgery for TA rupture. Higher levels of wound infection appear to be associated with open surgery [9,36,37]. However, higher rates of sural nerve injury have been reported with minimally invasive surgery [9,38]. Further research is needed to evaluate the long-term outcomes of various surgical techniques.

Limitations

This study has potential limitations and sources of biases, including the limitations of the included studies themselves. Due to reporting limitations in the included studies, we were not able to analyze demographic factors, which could influence the results of the measured outcomes. The incidence of complications could be affected by different treatment protocols. Inherent to systematic reviews of this nature, sample sizes among included studies vary greatly, which can be a cause of bias. In this systematic review, Fell et al.’s study accounts for a large proportion of the patients included [16]. Finally, various types of surgical procedures were used across the included studies, making a somewhat heterogeneous group. The degree of heterogenicity was not quantified in this review.

## Conclusions

Surgical repair of TA rupture demonstrates excellent long-term outcomes, including low rates of re-rupture and revision over a minimum of five years. While complications such as deep vein thrombosis and wound infections are observed, this systematic review highlights the reliability, durability, and overall success of surgical intervention in effectively treating this condition.

## Appendices

**Table 4:** Summary of search strategy

No.	Query	Last run via	Results
1	("Achilles tendon*" or "heel cord*" or "calcanea tendon*").mp.	Ovid Medline	13,178
2	exp Achilles Tendon/	Ovid Medline	9,644
3	1 or 2	Ovid Medline	13,178
4	(surger* or operation* or operating).mp.	Ovid Medline	3,653,733
5	exp Orthopedic Procedures/	Ovid Medline	363,740
6	exp Surgery, Plastic/	Ovid Medline	28,347
7	4 or 5 or 6	Ovid Medline	3,721,849
8	outcome*.mp.	Ovid Medline	3,241,035
9	exp Patient Outcome Assessment/ or exp Outcome Assessment, Health Care/	Ovid Medline	1,353,053
10	exp Postoperative Complications/	Ovid Medline	616,046
11	8 or 9 or 10	Ovid Medline	3,729,068
12	(tear* or rupture* or strain*).mp.	Ovid Medline	1,370,603
13	exp Tears/	Ovid Medline	10,900
14	12 or 13	Ovid Medline	1,370,603
15	3 and 7 and 11 and 14	Ovid Medline	1,249

No.	Query	Last run via	Results
1	('achilles tendon*':ti,ab,kw OR 'heel cord*':ti,ab,kw OR 'calcanea tendon*':ti,ab,kw OR 'achilles tendon'/exp) AND (surger*:ti,ab,kw OR operation*:ti,ab,kw OR operating:ti,ab,kw OR 'orthopedic surgery'/exp OR 'plastic surgery'/exp) AND (outcome*:ti,ab,kw OR 'adverse outcome'/exp OR 'outcome assessment'/exp OR 'postoperative complication'/exp) AND (tear*:ti,ab,kw OR rupture:ti,ab,kw OR strain*:ti,ab,kw OR 'rupture'/exp) NOT [medline]/lim	Embase	329

No.	Query	Last run via	Results
1	("Achilles tendon" or "heel cord" or "calcanea tendon" OR "Achilles tendons" OR "heel cords" OR "calcanea tendons"):ti,ab,kw	Cochrane Library	1,108
2	MeSH descriptor: [Achilles Tendon] explode all trees	Cochrane Library	440
3	#1 OR #2	Cochrane Library	1,108
4	(surger* or operation* or operating):ti,ab,kw	Cochrane Library	305,131
5	MeSH descriptor: [Orthopedic Procedures] explode all trees	Cochrane Library	17,742
6	MeSH descriptor: [Surgery, Plastic] explode all trees	Cochrane Library	155
7	#4 OR #5 OR #6	Cochrane Library	307,851
8	(recurren* or revision* or recurring or revising or reoperat* or "re-operat*" or complicat*):ti,ab,kw	Cochrane Library	317,823
9	MeSH descriptor: [Postoperative Complications] explode all trees	Cochrane Library	50,182
10	MeSH descriptor: [Reoperation] explode all trees	Cochrane Library	2,392
11	#8 OR #9 OR #10	Cochrane Library	337,920
12	#3 AND #8 AND #11	Cochrane Library	212
